# The role and underlying mechanism of miR-1299 in cancer

**DOI:** 10.2144/fsoa-2021-0014

**Published:** 2021-03-02

**Authors:** Deng Kaiyuan, Huang Lijuan, Sun Xueyuan, Zang Yunhui

**Affiliations:** 1 Department of Laboratory Medicine, The Second Affiliated Hospital of Harbin Medical University, 150086, China

**Keywords:** apoptosis, cancer, invasion, microRNAs, miR-1299, molecular mechanism, oncogene, proliferation, target protein, tumor suppressor

## Abstract

A type of evolutionarily conserved, noncoding, small, endogenous, single-stranded RNA, miRNAs are widely distributed in eukaryotes, where they participate in various biological processes as critical regulatory molecules. miR-1299 has mainly been investigated in cancers. miR-1299 is a tumor suppressor that regulates the expression of its target genes, activating or inhibiting the transcription of genes regulating biological activities including cell proliferation, migration, survival and programmed cell death. miR-1299 has become a hotspot in research of disease mechanisms and biomarkers; elucidation of the regulatory roles of miR-1299 in tumorigenesis, proliferation, apoptosis, invasion, migration and angiogenesis may provide a new perspective for understanding its biological functions as a tumor suppressor.

## Lay abstract

As key regulatory molecules, microRNAs participate in various biological processes and have become a widespread research focus. This article discusses how the microRNA miR-1299 plays a role as a tumor suppressor and participates in the regulation of tumor pathogenesis. We provide an overview of the role of miR-1299 in tumor diseases and discuss the pathogenesis and regulation mechanisms of miR-1299 in different specific cancers.

Cancer, cardiovascular diseases and infectious diseases are the three leading causes of death worldwide [1]. They seriously threaten health and continue to have a high incidence, leading to high morbidity and high mortality worldwide. At present, much research on the diagnosis, treatment and prognosis of cancer is focused on genetic and epigenetic factors. Epigenetic regulation is based primarily on DNA methylation, histone posttranslational modifications and noncoding RNAs, especially miRNAs.

miRNAs are rapidly emerging and potentially important entities in diseases such as cancers and cardiovascular diseases [2,3]. miRNAs consist of 19–24 nucleotides and are evolutionarily conserved noncoding small RNA molecules. Researchers have shown that they function mainly through binding to the 3′ untranslated region (3′-UTR) of the mRNA of their target genes to promote the degradation of transcripts or inhibit their normal translation. Thus they regulate expression posttranscriptionally and pretranslationally, affecting biological processes such as cell proliferation, apoptosis, invasion and migration [4,5]. Importantly, miRNAs can be potential tumor suppressors or oncogenes [6].

miRNAs can be divided into oncogene and tumor suppressor miRNAs [7]. As oncogenes, they cause cancer by targeting antiproliferative, anti-cell differentiation and proapoptotic genes, and cancer cells are thus modified to promote cell survival, the cell cycle and the expression of proliferative genes; as tumor suppressors, the expression of some miRNAs is reduced during cancer [8]. Interestingly, miRNAs can play different roles as carcinogens or tumor suppressors, depending on the target gene(s) of the miRNAs and individual tumor differences [9]. Whether as oncogenes or tumor suppressor genes, miRNAs play a regulatory role in a variety of biological processes, which suggests that the regulatory effects of miRNAs may have a potential role in cancer diagnosis and molecular targeted therapy.

In the process of miRNA maturation, primary transcripts of miRNAs (pri-miRNAs, large) are synthesized by RNA polymerase II and then cleaved and processed by the enzymes Drosha and Dicer to form miRNAs. Next, the mature miRNA is assembled into an RNA-induced silencing complex which recognizes the target mRNA by complementary base pairing and degrades the target mRNA or inhibits its translation based on the degree of complementation. Hence gene expression is regulated at the posttranscriptional level (Figure 1) [10,11].

**Figure 1.**
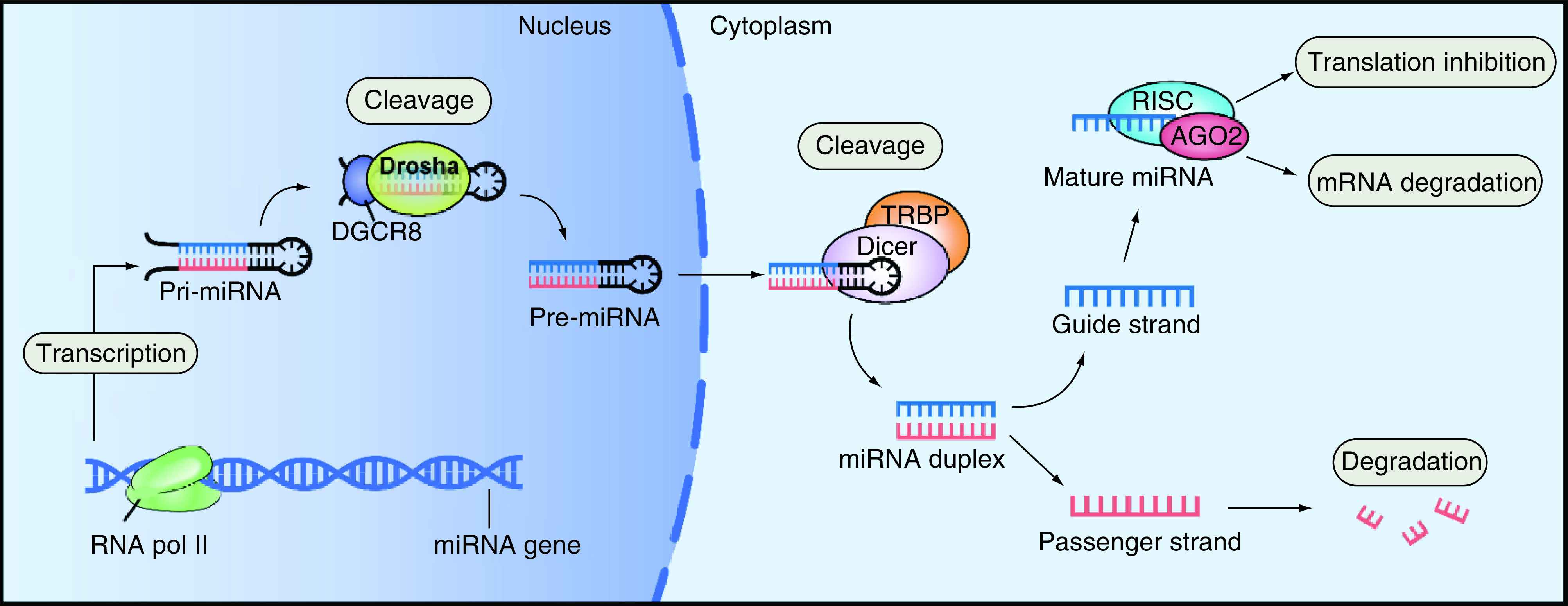
Biogenesis and function of miRNA.

Since 1993, when miRNAs were first discovered in nematode cells, many miRNAs have been found in animals, plants and viruses, and more than 500 miRNAs have been proven to exist in humans and other eukaryotes [12,13]. According to studies, fragile sites and genomic regions related to cancer are the most frequent locations of human miRNA genes [14]. Comparative genomic hybridization data from high-resolution arrays have shown that the copy number of miRNAs in human cancers is usually abnormal. Approximately 5300 miRNA target genes, as well as 52.5% of known miRNAs, are located in vulnerable genomic regions that are often modified in cancer [15]. Dysregulation of miRNAs may be attributed to various factors, including epigenetic alterations, genomic deletions, genomic amplification, retroviral insertion, mutagenesis, single nucleotide substitutions caused by mutations or single-nucleotide polymorphisms, as well as the direct activation or suppression of proteins [16]. Here we discuss the regulatory mechanism of dysregulated miRNAs in cancer and what biological functions they perform.

## miR-1299

miR-1299 is a tumor suppressor expressed in many tumor tissues [17–19]. Its expression levels in breast cancer, ovarian cancer, prostate cancer, colon cancer, cervical cancer, liver cancer, cholangiocarcinoma and colon cancer are lower than those in normal tissues. As a tumor suppressor, miR-1299 inhibits tumor cell proliferation, invasion and metastasis, improves chemotherapeutic sensitivity and regulates the development and progression of tumors. Moreover, the downstream targets of miR-1299 are complicated, indicating that it may regulate various target genes and consequently may play many roles in different disease mechanisms [20]. In recent years, there have been several studies on miR-1299, all of which have focused on the pathogenesis and therapeutic targets of cancer (Table 1). In this review, we summarize the mechanisms of miR-1299 in cancer-related biological processes including proliferation, apoptosis, angiogenesis, drug resistance, invasion and metastasis. This information will further explore the role of miR-1299 in the molecular mechanism of disease pathogenesis and can be used for disease diagnosis and treatment.

**Table 1.** The expression, targets, biological functions and role of miR-1299 in cancer.

Cancer type	Expression	Site	Target	Biological function	Role	Ref.
ESCC	Down	Tissue	EGFR-Akt-mTOR	Promote autophagy	Tumor suppressor	[21]
Breast cancer	Down	Tissue and cell	CDK6	Repress breast cancer proliferation, migration and invasion	Tumor suppressor	[22]
Cholangiocarcinoma	Down	Tissue and cell	–	Repress cell growth and metastasis	Tumor suppressor	[23]
Prostate cancer	Down	Tissue and cell	NEK2	Inhibit proliferation, invasion and migration of Pla cells	Tumor suppressor	[24]
Gastric cancer	Down	Tissue and cell	ETS1	Decrease the viability of GC cells, increase the rate of apoptosis	Tumor suppressor	[25]
TNBC	Down	Tissue and cell	MMP	Inhibit cell migration and invasion of TNBC	Tumor suppressor	[26]
Melanoma	Down	Tissue and cell	ARG2 tyrosinase	Promote autophagy	Tumor suppressor	[27]
Colon cancer	Down	Tissue and cell	STAT3	Inhibit colon cancer cell growth	Tumor suppressor	[28]
HCC	Down	Tissue and cell	CDK6	Inhibit cell proliferation	Tumor suppressor	[29]
Ovarian cancer	Down	Tissue and cell	NOTCH3	Induce cardiomyocytes apoptosis	Tumor suppressor	[30]
Cervical cancer	Up	PI003-induced apoptosis of cells	P1M1	Promote cell apoptosis	Tumor suppressor	[31]

ESCC: Esophageal squamous cell carcinoma; HCC: Hepatocellular carcinoma; TNBC: Triple-negative breast cancer.

## miR-1299 & cancers

Research has shown that many miRNAs function as regulators of tumor cells to affect their proliferation and apoptosis by regulating downstream genes and participating in complex signal transduction networks in both the cells and the tumor microenvironment [32]. In recent years, research on the role of miRNAs in cancer has focused on carcinogenesis, genomic instability, cell proliferation and apoptosis, cancer cell replication potential, angiogenesis regulation, the immune response, tumorigenesis and development, tumor invasion and metastasis, drug resistance and prognosis [33,34].

**Figure 2.**
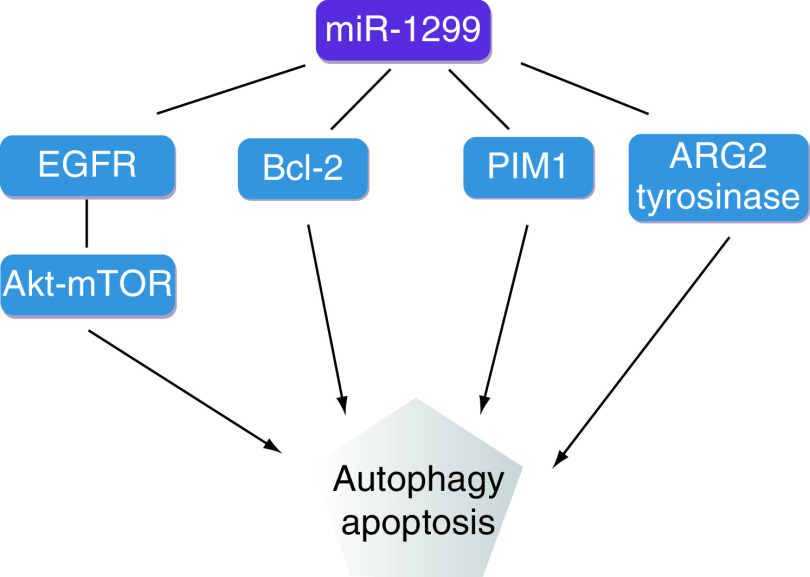
miR-1299 promotes autophagy and apoptosis of tumor cells by targeting EGFR-Akt-mTOR, Bcl-2, PIM1 and ARG2-tyrosinase.

**Figure 3.**
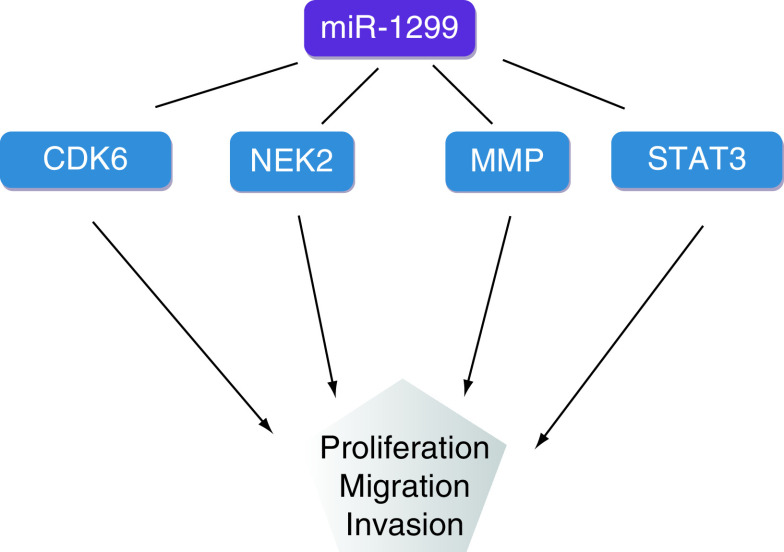
miR-1299 inhibits tumor cell proliferation, migration and invasion by targeting *CDK6*, *NEK2*, *MMP* and *STAT3*.

Analysis and evaluation of the diagnosis and prognosis of various tumors has revealed that miR-1299 plays a significant role in tumors. miR-1299 regulates various target genes, such as *NEK2*, *MMP*, *STAT3*, *PIM1*, *CDK6*, *EGFR*, *ARG2* and *BCL2*, to play a role as a tumor suppressor gene in a variety of cancers (Figures 2 & 3) [35–37].

### miR-1299 & breast cancer

Breast cancer is the predominant malignant tumor that endangers women’s health, and it ranks second in cancer mortality among women worldwide [38]. The breast cancer incidence among women is at least 15%, and its incidence is increasing [39]. The malignant proliferation and metastasis of breast epithelial cells can seriously endanger the lives of patients [40], but the etiology of breast cancer is not fully understood; therefore the pathogenesis and potential treatment of breast cancer needs further study.

Liu *et al.* [22] studied the development and progression of breast cancer and showed that miR-1299 was downregulated in breast cancer tissues compared with healthy breast tissues. In addition, RT-PCR results showed that its expression levels in the breast cancer cell lines MCF7 and BT474 were downregulated, and Transwell assays with or without Matrigel confirmed that its downregulation increased the proliferation and migration of MCF7 and BT474 cells. miR-1299 contains a hsa_circ_0136666 binding site; thus miR-1299 may function as a downstream RNA of hsa_circ_0136666 to act as a tumor suppressor in breast cancer cells. Overexpressed miR-1299 suppresses breast cancer cell proliferation, migration and invasion. Moreover, miR-1299 inhibits cell cycle progression by binding to the transcript of one of its targets, *CDK6*. These results indicate that miR-1299 can act as a tumor suppressor in breast cancer cells and regulate the growth and metastasis of breast cancer through the hsa_circ_0136666/miR-1299/CDK6 axis.

Studies have found that paclitaxel (PTX) is useful in the chemotherapy of breast cancer [41]. It blocks cell cycle progression and induces apoptosis to inhibit tumor cell growth [42]. To reveal the function of miR-1299 in PTX-resistant breast cancer cells, Zhang *et al.* [43] conducted loss-of-function assays. The results showed that miR-1299 is regulated by the upstream molecular sponge circ_0006528; knocking down circ_0006528 increased the expression of miR-1299, which decreased the IC_50_ value of PTX as well as hindering cell proliferation, migration, invasion and autophagy and induced apoptosis in breast cancer cells *in vitro*. Interestingly, miR-1299 is able to target the 3′-UTR of *CDK8*; *in vivo* experiments showed that circ_0006528 knockdown downregulated* CDK8* expression via reducing the sponging of miR-1299. This pathway therefore delays cell cycle progression, inhibits the growth of PTX-resistant tumors and promotes breast cancer cell apoptosis.

In the study by Sang *et al.* [26], expression levels of miR-1299 in 164 samples of triple-negative breast cancer (TNBC) were investigated. The results showed that lower expression of miR-1299 was related to the migration and invasion of TNBC cells. As miR-1299 is a tumor suppressor, its downregulation maintains the high migration and invasion characteristics of TNBC cells. In order to further study the regulation mechanism of miR-1299 in TNBC, the authors performed luciferase analysis and showed that* MMP* is the downstream target protein of miR-1299; RT-PCR results showed that miR-1299 can target and regulate the expression of* MMP* family members. On this basis, miR-1299 was found to inhibit the migration and invasion of TNBC cells at least in part by inhibiting the expression of MMPs, providing new potential therapeutic targets for TNBC treatment.

### miR-1299 & cervical cancer

Cervical cancer is the third most common cancer in women worldwide [44], and concepts and knowledge about its prevention and treatment are rapidly evolving. It develops through persistent infection with high-risk human papilloma virus and is a leading cause of death among women worldwide [45]. The incidence of cervical cancer across the globe has been reduced through regular surveillance via human papilloma virus and Pap smear-based testing. However, considerable disparities exist in the occurrence and outcome of cervical cancer in various populations [46].

*PIM* proteins are a family of short-lived serine/threonine kinases that are highly evolutionarily conserved in multicellular organisms [47]. Unlike the activities of other kinases, the activity of* PIM* kinases is not primarily regulated by phosphorylation; instead,* PIM* kinases are mainly regulated by transcription [48]*. PIM* family members are weak oncogenes but can contribute to tumorigenesis by selectively enhancing tumorigenic capabilities [49]. Given their overexpression in many cancers and involvement in cancer-specific pathways, these kinases are of interest as drug targets [50]. *PI003*, as a novel synthesized pan-PIM inhibitor, could induce the death-receptor and mitochondrial apoptosis involved in some miRNA regulation, and also possesses remarkable antitumor activity and apoptosis-inducing capacity *in vivo* [51]. Liu *et al.* [31] found that* PI003* induces apoptosis via the death-receptor and mitochondrial pathways in HeLa cells. The authors used miRNA microarray analysis to identify miRNAs expressed in HeLa cells when* PI003* induced apoptosis. The results showed that miR-1299 was upregulated during PI003-induced apoptosis in HeLa cells compared with control cells and that a miR-1299 mimic markedly decreased the expression level of *PIM1*, suggesting that miR-1299 negatively regulates *PIM1* and possesses remarkable antitumor activity and apoptosis-inducing effects *in vitro*.

### miR-1299 & cholangiocarcinoma

Cholangiocarcinoma (CCA) is a highly malignant cancer of the digestive system with high mortality worldwide. Currently, no treatment regimens active against this disease have been developed [52,53]. CCA originates from tumorigenic transformation of highly malignant biliary epithelial cells. Its 5-year overall survival rate is less than 40% [54]. Due to limited detection techniques, patients with uncured CCA in early stages contribute significantly to the increased mortality of the disease. Despite significant advances in clinical surgical techniques and neoadjuvant chemoradiotherapy, the prognosis of patients with CCA remains poor [55]. Hence, studies of the mechanisms involved in the development and progression of CCA are needed.

A study of 58 newly diagnosed CCA patients was performed by Xu *et al.* [23]. The data from qRT-PCR revealed that circ_0005230 expression was enhanced in CCA specimens compared with healthy tissues. After validating the elevation of circ_0005230 in CCA specimens, its clinical implication was investigated; analysis indicated that circ_0005230 expression is related to larger tumor size, positive lymph node invasion and advanced tumor node metastasis stages for CCA patients. In order to further study the pathogenesis of CCA, the authors performed luciferase analysis and showed that there is a binding site between circ_0005230 and miR-1299. RT-PCR showed that the upregulated circ_0005230 can significantly inhibit the expression of miR-1299. After confirming that circ_0005230 could interact with miR-1299, it was imperative to evaluate whether the oncogenic activities of circ_0005230 could be attributed to its negative regulation by miR-1299. Both functional assays and *in vivo* research experiments showed that si-circ_0005230 cotransfected with an miR-1299 inhibitor partially rescued the tumor-suppressing effects of si-circ_0005230 in CCA cells. In general, miR-1299 inhibits the proliferation and migration of CCA tumor cells by negatively regulating its molecular sponge circ_0005230, thereby exerting a tumor-suppressive effect.

### miR-1299 & esophageal squamous cell carcinoma

Esophageal cancer is a common digestive tract tumor, ranking sixth in global cancer mortality, and China is a high-incidence area [56]. Esophageal squamous cell carcinoma (ESCC), the primary histological type of esophageal cancer, accounts for more than 90% of esophageal cancer cases [57]. Treatments for ESCC, including surgery, radiotherapy and chemotherapy, have significantly increased the survival rate of ESCC patients; however, the development of the prognosis still needs to be resolved [58]. Therefore there is an urgent need to identify relevant molecular targets for the development of new treatments for ESCC [59]. The growth and progression of ESCC is a complex pathological process. Various oncogenes have been demonstrated to participate in the pathogenesis of ESCC. However, accumulating research has shown that other types of biomolecules, such as noncoding RNAs, are also involved in this pathogenesis [60].

Meng *et al.* [21] found that miR-1299 is expressed at low levels in ESCC tissues compared with normal tissues and that it can inhibit the autophagy of ESCC cells induced by starvation or rapamycin. In order to further study the regulation mechanism of miR-1299, dual luciferase reporter gene detection showed that miR-1299 significantly reduced the luciferase activity of *EGFR* 3′-UTR. At the same time, the *EGFR* mRNA expression level detected by qRT-PCR showed that, miR-1299 significantly reduces the expression of *EGFR*. miR-1299 has a binding sequence in the 3′-UTR of *EGFR* that regulates the downstream Akt-mTOR pathway, thereby promoting the autophagy of ESCC cells*. EGFR* is a carcinogenic tyrosine kinase that promotes the proliferation, differentiation, metastasis and angiogenesis of tumor cells and directly regulates tumor cell autophagy [61]. In this study, the author proposed the existence of a ciRS-7/miR-1299/EGFR pathway. During autophagy of ESCC cells induced by rapamycin, ciRS7 acts as a molecular sponge of miR-1299, reducing the expression of miR-1299 and upregulating *EGFR* to inhibit the autophagy of ESCC cells. This finding provides a basis for miR-1299 to act as a tumor suppressor to regulate ESCC.

### miR-1299 & liver disease

Hepatocellular carcinoma (HCC) ranks among the top five causes of cancer-related death worldwide [62]. Its pathogenesis is based on long-term liver injury, inflammation and regeneration, and no treatments with favorable curative effects have been identified to date [63]. The survival rate of HCC patients remains low, and its incidence and mortality are increasing [64,65]. Exploring the molecular mechanisms is essential for improving the treatment, diagnosis and prognosis of hepatocellular carcinoma.

Zhu *et al.* [29] reported that miR-1299 was expressed at lower levels in HCC cells than in normal hepatocytes, and MTT assays showed that miR-1299 overexpression inhibited HCC cell proliferation. Cell cycle analysis showed that the downregulation of miR-1299 reduced the number of cells in G0/G1 phase and increased the number of cells in S phase. Researchers also found that the cell cycle regulator* CDK6 i*s a target of miR-1299, and that miR-1299 can bind to the 3′-UTR of *CDK6*. The simultaneous downregulation of *CDK6* and miR-1299 increased the proliferation ability of HCC cells, indicating that miR-1299 inhibits the proliferation of HCC cells by downregulating the expression of *CDK6*.

Yu *et al.* [66] found that compared with the normal control group, miR-1299 was significantly downregulated in HCC cells and tissues and that its inhibitory effect promoted the proliferation, cell cycle progression, migration and invasion of HCC cells. In terms of the mechanism by which miR-1299 affects the function of HCC cells, their study further confirmed that circMAST1 could be used as a molecular sponge for miR-1299. Via a WST-1 assay, they found that the proliferation and colony-forming abilities of HCC cells cotransfected with siRNA-circMAST1 and miR-1299 inhibitor were higher than those of HCC cells transfected only with siRNA-circMAST1. At the same time, they revealed a negative correlation between miR-1299 and *CTNND1* expression levels; miR-1299 inhibition significantly increased* CTNND1* protein levels, and miR-1299 mimics reduced the expression of *CTNND1* to promote *CTNND1-*induced proliferation and invasion of HCC cells. The above results indicate that miR-1299 can be used as a tumor suppressor to inhibit the proliferation and migration of liver cancer cells.

### miR-1299 & gastric cancer

Gastric cancer (GC) is one of the most aggressive malignant neoplasms of the digestive system. Its mortality rate is as high as 12%, and it is currently ranked third in mortality among malignant diseases [67,68]. Owing to the lack of sensitive and specific screening methods, over 80% of patients are diagnosed with advanced GC and extensive lymph node metastasis as well as distant metastasis [69]. In recent years, even though the prevalence of GC has been well controlled, the prognosis of GC patients has remained poor [70].

A previous study showed that miR-1299 expression in GC cells is significantly reduced compared with that in normal cells. Interestingly, low expression of miR-1299 can increase the viability of GC cells, increase the number of cell colonies, decrease the apoptosis rate and accelerate progression of the cell cycle. In addition, this effect can be due to feedback regulated by *ETS1.* Through utilization of the bioinformatics tool TargetScan database, it was discovered that *ETS1* possesses predicted binding sites with miR-1299. qRT-PCR assay went on to clarify that oncogenic *ETS1* expression was overtly upregulated in GC cells compared with normal cells. Hence*, ETS1 w*as selected for further study, and luciferase reporter gene detection showed that it directly binds to miR-1299. In addition, the ectopic expression of miR-1299 resulted in a decrease in the expression of *ETS1* at the mRNA and protein levels, which proved that *ETS1* is the downstream target of miR-1299. Thus miR-1299 can act as a tumor suppressor of GC through the miR-1299/ETS1 pathway [25].

### miR-1299 & prostate cancer

Prostate cancer (PCa) is a malignant tumor with a high incidence in men. In 2012, approximately 1.1 million people were diagnosed with PCa worldwide [71]. Although technologies for the screening, diagnosis and treatment of PCa have improved significantly, the disease still threatens people’s lives seriously [72,73], mainly because PCa is a typically heterogeneous disease with an insidious onset and slow progression. However, as the disease progresses, metastasis to bones or other organs may occur, which eventually leads to relatively high mortality [74].

Zhang *et al.* [24] determined the expression of miR-1299 in 35 PCa tissues and adjacent tissues as well as in a PCa cell line (PC-3) and prostate epithelial cell line (RWPE-1) by qRT-PCR. It was confirmed that the expression level of miR-1299 was substantially decreased in PCa tissues compared with adjacent tissues. In addition, the expression of miR-1299 in PC-3 cells was also substantially downregulated compared with that in RWPE-1 cells. Luciferase reporter gene assays demonstrated that overexpression of miR-1299 significantly decreased the luciferase activity of wild-type* NEK2*, further verifying that *NEK2* is a target gene of miR-1299*. NEK2* is a member of the cell cycle-regulating protein kinase family, a centrosome-related protein kinase whose expression is abnormally elevated in multiple tumors. Furthermore, abnormality of *NEK2* has been found to be linked to the incidence and development of many malignant tumors [75]. Subsequently, the authors detected the expression of *NEK2* in PCa tissues and cells. As expected, the expression of *NEK2* in PCa tissues and cells was significantly higher than in the control groups. To confirm the effect of the miR-1299/NEK2 pathway on the proliferation and migration of prostate cancer cells, MTT assays, flow cytometry and Transwell assays were conducted; overexpression of miR-1299 was found to significantly inhibit the expression of *NEK2*, reduce the relative viability of PCa cells and reduce their proliferation and migration rates. These results indicate that miR-1299 is a novel tumor suppressor in PCa through its negative regulation of *NEK2*.

### miR-1299 & melanoma

Human skin diseases such as chloasma and melanoma are caused by skin aging and pigmentation [76]. The synthesis of melanin, the transfer of melanosomes to keratinocytes and the degradation of melanosomes in melanocytes are significant factors leading to skin pigmentation [77].

Kim *et al.* [27] found that miR-1299 is downregulated in melanin deposition diseases, including chloasma, and its expression level is inversely proportional to that of *ARG2*. *ARG2* is expressed in diverse extrahepatic tissues lacking a complete urea cycle, including skin keratinocytes and fibroblasts, and can reduce the degradation of melanosomes to increase pigmentation [78]. These results showed that miR-1299 plays an inhibitory role in melanin production and that it targets *ARG2* to reduce melanin degradation by alleviating the inhibition of aging-induced autophagy to enhance pigmentation in melanoma.

### miR-1299 & ovarian cancer

Ovarian cancer (OC) is one of the deadliest malignant tumors in women and has a low survival rate. In the USA, OC has the sixth highest cancer-related mortality rate [79]. The main reason for this phenomenon is its late-stage diagnosis and high tumor recurrence rate. Approximately 60% of patients with advanced disease experience relapse after treatment, and the cure rate after relapse is extremely low [80–82].

Pei *et al.* [30] measured the level of mature miR-1299 in 35 fresh OC tissues, 16 normal ovarian tissues and 4 OC cell lines. Compared with the level in normal tissues, the expression of miR-1299 in OC tissues was significantly downregulated, and the authors found that tumor differentiation was closely related to this downregulation. Studies have shown that miR-1299 can inhibit cell proliferation, colony formation and 5-ethynyl 2′-deoxyuridine (EdU) incorporation and induce G0/G1 cell cycle arrest in OC cells. After transfection into a constructed ovarian cancer model via liposomes, miR-1299 significantly reduced the tumor volume and weight, confirming the tumor-suppressive function of miR-1299 in OC tumorigenesis. The authors also proposed that miR-1299 is a novel negative regulator of *NOTCH3*, which is downregulated in OC. In addition, the expression of *NOTCH3* was found to be elevated in OC and closely correlated with the clinical stage, pathological grade, lymph node metastasis, drug-resistant recurrence and survival rate [83].* NOTCH3* promotes the malignant progression of OC through enhancement of tumor cell proliferation, stemness maintenance and apoptosis resistance [84]. Overexpression of miR-1299 was found to play a tumor-suppressive role both *in vitro* and *in vivo* by partially inhibiting cell proliferation. The lncRNA TUG1 acts as a sponge for miR-1299 and promotes cell proliferation by upregulating *NOTCH3*. TUG1 is also a potential target for *NOTCH3*, forming a miR-1299/NOTCH3/TUG1 feedback loop to regulate the proliferation and apoptosis of ovarian cancer cells.

Through microarray and qRT-PCR analyses, Xia *et al.* [85] identified that miR-1299 is dramatically downregulated in OC samples, and this was correlated with PTX resistance. The proposed mechanism was that circTNPO3 acted as a sponge for miR-1299, and downregulation of circTNPO3 significantly promoted miR-1299 expression. As with the studies in PCa discussed above, *NEK2* was revealed to be a target gene of miR-1299 [84]. Functionally, knockdown of circTNPO3 enhanced cell sensitivity to PTX by promoting PTX-induced apoptosis *in vitro* and *in vivo* by upregulating the expression of miR-1299. The results of rescue assays indicated that downregulation of miR-1299 partially reversed the inhibitory effect of circTNPO3 silencing on OC cell proliferation, indicating that miR-1299 is involved in circTNPO3-mediated chemoresistance in OC. Subsequently, functional assays illustrated that the miR-1299/NEK2 axis inhibits the carcinogenic effect of circTNPO3. In conclusion, circTNPO3 contributes to the PTX resistance of OC cells at least partially through upregulating *NEK2* expression by sponging miR-1299. The circTNPO3/miR-1299/NEK2 signaling pathway might play vital roles in the tumorigenesis and chemoresistance of OC.

Zhao *et al.* [86] also confirmed that miR-1299 was downregulated in OC tissues and that downregulation of miR-1299 promoted OC cell proliferation, migration and invasion. More importantly, miR-1299 inhibitors rescued the proliferation, migration and invasion of OC cells via silencing of its molecular sponge RHPN1-AS1.

### miR-1299 & colon cancer

Colon cancer is the most common primary malignant tumor of the digestive system [87] and the third most commonly diagnosed cancer worldwide [88]. Colon cancer can easily recur, making it a devastating disease with an extremely low survival rate [89].

A study by Wang *et al*. [28] demonstrated, by analyzing and comparing the tissues of 60 colon cancer patients with adjacent normal tissues, that miR-1299 was significantly downregulated, inhibited tumor size and was correlated with tumor node metastasis stage in colon cancer. Clinical data and experimental models published by Kamran *et al*. indicated that STAT3 plays essential roles in colon cancer and can regulate cell growth by promoting cell proliferation and inhibiting apoptosis [90]. To further study the regulatory mechanism of miR-1299 in colon cancer, a luciferase reporter gene assay was performed; the report stated that miR-1299 inhibited the expression of *STAT3* at the transcriptional level. On the other hand, the researchers found by real-time PCR that the expression of miR-1299 was negatively correlated with the expression of *STAT3* in colon cancer tissue: overexpression of miR-1299 can significantly inhibit the expression of *STAT3*, and when the expression of miR-1299 decreases, the expression of *STAT3* increases, which is further evidence that miR-1299 can regulate *STAT3*. Annexin V-FITC staining showed that apoptosis of colon cancer cells was stimulated when miR-1299 was overexpressed. In contrast, apoptosis of colon cancer cells was clearly decreased when the expression of miR-1299 was inhibited. The above research confirmed that miR-1299 can inhibit the proliferation and promote the apoptosis of colon cancer cells by reducing the expression of *STAT3*, the phosphorylation level of* STAT3* and the expression of its downstream proteins [28].

## Clinical significance

The proliferation and apoptosis of cancer cells play an important role in the occurrence and development of cancer. A defining hallmark of cancer is aberrant cell proliferation [91]. Aberrations in the regulation of a restricted number of key pathways that control cell proliferation and cell survival are a prerequisite for the establishment of all tumors. Suppressed apoptosis combined with a deregulation of cell proliferation is the minimal common platform [92].

Apoptosis is a cellular process regulated by different groups of executioner and regulatory molecules, and their aberrant function is fundamental to the growth of tumors and the development of anticancer drug resistance [93]. A disruption in the balance of pro- and anti-apoptotic proteins contributes to carcinogenesis by reducing the apoptosis of malignant cells. For example, disequilibrium between pro- and anti-apoptotic* BCL2* proteins can promote cancer cell survival [94]. Therefore apoptosis has become one of the prime molecular targets for drug discovery and development, particularly for cancer.

There is evidence that dysregulation of miRNAs is associated with different human cancers and that miRNAs can function as oncogenes and tumor suppressors [95]. Their modulation (overexpression or downregulation according to the specific miRNA) in cancer cells often sensitizes cells to apoptotic and antiproliferative treatments and then participates in regulating cell apoptosis and proliferation, which suggests that targeting miRNAs to modulate apoptosis or the proliferation of cancer cells can be utilized for cancer treatment [96].

In recent years, many research efforts regarding miR-1299 have been carried out in various cancers. In the occurrence and development of tumors, the expression of miR-1299 is downregulated, and it plays a considerable role in repressing tumorigenesis and blocking tumor progression. As a tumor suppressor, miR-1299 is considered to be involved in the regulation of tumorigenesis. In constructing mouse models of ovarian cancer, upregulating the expression of miR-1299 can inhibit tumor growth and lead to tumor cell growth cycle arrest [30]. In the tissues of patients with prostate cancer, low expression of miR-1299 leads to a decrease in tumor proliferation and migration rate, which inhibits tumor growth [24]. In addition, high miR-1299 levels in tumor cells can lead to high levels of prolactin synthesis and secretion and poor control of serum prolactin levels [97]. In these tumor patients, miR-1299 does exert antitumor activity by regulating different genes. As a tumor suppressor, although there is less information on miR-1299, it is worth exploring as a potential tumor treatment.

## Conclusion & future perspective

With the continuous development of bioinformatics, the ways in which miR-1299 is involved in the development and progression of tumors need to be explored further to better understand the detailed mechanisms of miR-1299 and related genes. As a tumor suppressor, miR-1299 targets multiple relevant genes and forms several signaling pathways to exert its synergistic effects. It is involved in the regulation of multiple aspects of cancer, including cell proliferation, migration, apoptosis, invasion and tumorigenesis. Thus miR-1299 is a promising biomarker for early diagnosis and prognosis of tumors, providing novel, safe and effective insights into future molecular targeted therapies and bringing new hope to cancer patients. However, current research on miR-1299 is limited to tumors. In the next 5–10 years, the mechanism of miR-1299 in other diseases, such as cardiovascular and endocrine diseases, also merits further research, to study whether miR-1299 participates in cell apoptosis, proliferation, migration and so on in other diseases by regulating particular genes. By studying the regulation mechanism of miR-1299, we can predict whether miR-1299 can be involved in regulating the pathological mechanisms of other diseases in addition to being a tumor suppressor.

**Executive summary**

- Cancer is a disease with high morbidity and high mortality worldwide.
- Recently, many studies on the diagnosis, treatment and prognosis of cancer have focused on genetic and epigenetic factors, especially miRNAs.
- miRNAs can play different roles as carcinogens or tumor suppressors, depending on the target gene(s) of the miRNAs and individual tumor differences.
- These two ‘types’ of miRNAs are associated with multiple biological processes, such as tumor proliferation, apoptosis, invasion and migration, suggesting that the regulatory effects of miRNA may have a potential role in cancer diagnosis and molecular targeted therapy.
- miR-1299 is abnormally expressed in many tumor tissues.
- miR-1299 could be used as a tumor suppressor to regulate tumor cell proliferation, invasion and metastasis, improve chemotherapy sensitivity and regulate tumor development and progression.
